# From staff-mix to skill-mix and beyond: towards a systemic approach to health workforce management

**DOI:** 10.1186/1478-4491-7-87

**Published:** 2009-12-19

**Authors:** Carl-Ardy Dubois, Debbie Singh

**Affiliations:** 1 University of Montreal, Faculty of Nursing Sciences, CP 6128 - succursale Centre-ville Montréal, Québec, H3C 3J7, Canada; 2 Health Services Management Centre, University of Birmingham Edgbaston, Birmingham, B15 2RT, UK

## Abstract

Throughout the world, countries are experiencing shortages of health care workers. Policy-makers and system managers have developed a range of methods and initiatives to optimise the available workforce and achieve the right number and mix of personnel needed to provide high-quality care. Our literature review found that such initiatives often focus more on staff types than on staff members' skills and the effective use of those skills. Our review describes evidence about the benefits and pitfalls of current approaches to human resources optimisation in health care. We conclude that in order to use human resources most effectively, health care organisations must consider a more systemic approach - one that accounts for factors beyond narrowly defined human resources management practices and includes organisational and institutional conditions.

## Background

Health care systems' ability to provide safe, high-quality, effective, and patient-centred services depends on sufficient, well-motivated, and appropriately skilled personnel operating within service delivery models that optimise their performance[1,2]. However, both developing and developed countries are experiencing shortages in health care human resources. Two recent major reports have estimated the global shortage at more than four million workers [3,4]. Sub-Saharan countries, for example, must nearly triple their current number of workers if they are to progress towards achieving the health Millennium Development Goals. Meanwhile, analysts project that the shortage of registered nurses in the United States (US) could reach as high as 500 000 by 2025 [5], with a projected deficit of 200 000 physicians by 2020 [6]. This looming and global human resources (HR) crisis is the culmination of shortages of physicians, nurses, allied professionals, support workers and administrators. It is also affected by factors such as societal trends towards reduced work hours, workforce ageing, and early retirement (particularly in industrialised countries).

The policies and methods used to manage HR are at the core of any sustainable solution to health care system performance and can constrain or facilitate health care sector reform [7]. In developing countries, workforce imbalances have been identified as one of the main bottlenecks that compromise population health development. In developed countries, those imbalances are manifest amidst other concerns such as waiting lists, crowded emergency departments, understaffed wards, and a lack of time to provide patient-centred care [8,9]. These difficulties arise from quantitative imbalances and from inadequate approaches to HR management that may result in overusing, underusing, or misusing available health care personnel.

Health care organisations worldwide have been exploring innovative ways to deploy their workforces. There has been a focus on staff-mix, i.e. achieving a specific mix of different types of personnel, with an increasing interest in evidence about the value and contributions of different staff-mixes to patient, personnel, and organisational outcomes. Current evidence suggests that staff-mix cannot be considered in isolation from the contexts in which people work. In order to optimise HR, managers must extend beyond simple staff-mix modifications to address organisational and system factors.

To support planner, policy makers and workforce planners, this article reviews the main approaches to and limitations of conventional health care personnel deployment. We contend that the current staff-mix focus is both restrictive and static, and that it fails to account for staff members' skills and their effective utilisation. The second part of the article examines several options that offer a more dynamic solution that introduces the notion of skill management, referring to the mechanisms used by an organisation to optimise the utilisation of its workforce. These options emphasise enabling health care providers to practise to the full extent of their education, training, skills, knowledge, experience, and competence. We conclude by discussing levers that health care organisations and systems must mobilise to ensure that available personnel are used to their fullest potential.

## Methods

Our findings are based on a structured review of published literature, including articles, reviews, comparative studies, observational studies, and dissertation identified through a range of electronic databases: Medline, PubMed, Embase, Current Contents, CINAHL and Google Scholar. Other relevant materials (research reports, administrative reports, and articles) were collected through website searching, reference chaining and contacting experts in the field. The search focused on the literature between 1995 and 2008. However, some key literature prior to 1995 has been included when it was considered to be of particular relevance. The following key-words uncovered many hundreds of 'hits': staff-mix, skill-mix, human resource management, human resource optimisation, workforce performance, human capital, skill management, human resources for health, performance management. All references were reviewed by title and abstract to determine their potential relevance to the review. Letters, comments and editorials were systematically excluded. References that related directly to the subject matter in either the title or the abstract were selected for a more in depth review. In total, we examined full copies of 250 selected studies more thoroughly.

The evaluations of the studies and the data extraction were performed manually by the two investigators. Papers were first sorted into two categories: conceptual papers and empirical papers. Conceptual papers were evaluated and sorted according to their theoretical foundations, their comprehensiveness, their relevance and their contribution to subsequent work in the field. Empirical papers were evaluated and classed based on their relevance to the review objective and appropriate criteria of validity (research design, sampling and methods of analysis).

We used the technique of interpretative synthesis to collate the findings. This approach involved building a general interpretation grounded in the findings of separate studies and then integrating evidence from across the studies into a coherent theoretical framework comprising a network of constructs and the relationships between them [10]. As for the search strategy, the analysis focused first on evidence and theoretical perspectives drawn from the health care sector; however, as we advanced in the analysis, it has become evident that human resource management is a topic with diffuse boundaries that overlaps with several other fields. Although our selection of articles was clearly focused on human resources in health care, we had to extend our investigation to a wider range of literature in order to fill some gaps of evidence, gain insight from other areas and elaborate the emerging analysis. We particularly draw on theoretical perspectives and empirical work in sociology, economics, management, industrial and labour relations, and psychology that address different aspects of the domain of human resource management. Those works account for 20% of the 250 selected papers. The selection of articles, the extraction and the analysis therefore involved a constant dialectic and iterative process conducted concurrently with theory generation.

## Discussion

### Personnel deployment conceptualised as a staff-mix issue

Managing human resources in health care involves organising groups of workers with different professional backgrounds, skills, grades, qualifications, expertise and experience in order to achieve optimal patient care. This distinctive feature of health care has become more prominent during recent decades with the emergence of numerous new professions, specialties and occupations. These developments have drawn considerable attention to the concepts of staff-mix and skill mix as policy tools for developing the best combinations of skills across professions and organisations, as well as at the individual level. Increased interest in achieving optimal staff-mix also results from pressures arising from both the supply and demand sides of health care. On the supply side, changing the mix of health care staff has often been used as a resourcing strategy to address shortage problems. On the demand side, those changes have been implemented as a means to enlarging the scope of services, fill previously unmet health needs and improve patient care [11,12].

While many regard adequate staff and skill mix to be prerequisites for meeting patients' needs for high-quality care, HR adequacy is, in reality, hard to assess because it relates to many different parameters, including needs, preferences, availability, cost and quality. In this regard, recent reviews have highlighted the diversity of ways in which personnel deployment across teams and organisations is conceptualised [13-15]. Reviews suggest that although the concepts of staff-mix and skill mix are often used interchangeably, the four most prevalent conceptualisations are closer to the notion of staff-mix. We discuss these conceptualisations below.

#### Number of personnel

This conceptualisation focuses on the total number of workers in defined occupational groups. It takes into account the volume of work assigned to a given staff member or the amount of direct patient contact a worker experiences over a defined period of time. Common measurements are the number of hours of professional care per patient, per day; and the number of full-time equivalent workers per patient, per day. For pharmacists, the ratio has been defined as the number of prescription orders filled per day. For some physicians, the number of certain procedures performed per year is measured.

Research on personnel numbers has focused largely on nurses, and is based on the hypothesis that a lower nurse-to-patient ratio results in a greater workload and poorer quality of care due to time pressures that affect a person's ability to implement best-practice standards. Several empirical studies and systematic reviews support this hypothesis and indicate that the numbers of nurses in a unit and the number of nurses per patient affect patient outcomes, including adverse events, readmissions and mortality [16-22]. One study found that each additional patient in a typical nursing workload situation resulted in an average 7% increase in failure-to-rescue [23]. In another study, hospitals in which nurses cared for an average of eight patients each had risk-adjusted mortality rates following common inpatient surgical procedures that were 31% higher than hospitals in which nurses cared for four patients each [24]. Such findings have prompted legislation on safe staffing ratios for nurses in two jurisdictions: California and the state of Victoria in Australia. Yet, there is currently no clear-cut evidence of the effectiveness of such legislated ratios, which may prevent managers from making local decisions about appropriate staffing and are insensitive to many contextual factors (e.g., changes in patient dependency, presence of ancillary personnel or non-nurse providers, technology).

In contrast to nursing research, studies of physician resources are based on the premise that higher volumes, rather than hindering the ability to meet patients' needs, lead to improved experience and high-level technical skills [25]. Evidence from recent systematic reviews and observational studies suggests that higher volumes are, for physicians, associated with lower error rates and lower patient mortality rates [26-28]. Another study that used hospitals as the unit of aggregation showed that facilities with higher case volumes experienced lower complication rates [29]. Such positive findings are, however, balanced by some contradictory evidence. In controlling for institutional factors, some studies have failed to find that physicians who performed high rates of technical procedures experienced lower rates of adverse outcomes, suggesting that improved results reported in other studies may have been due to institutional rather than physician-specific factors [30-33].

#### Mixing qualifications

This conceptualisation focuses on the proportion of highly qualified staff members in the overall pool of professional resources. As yet, there is no indication of the appropriate ratio for any grade on the health care team, although several observational studies support the view that a rich mix of qualified personnel with advanced degrees or specialty certifications is associated with better clinical outcomes. Blegen et al [34] suggest that having a nursing team that is richer in registered nurses contributes to lower patient mortality rates. In a landmark study, Aiken et al [35] found an inverse relationship between the proportion of registered nurses holding undergraduate degrees and patient mortality rates within 30 days of admission: a 10% increase in the proportion of nurses with undergraduate degrees was associated with a 5% decrease in the likelihood of patients dying. Another study found that people cared for in the community by undergraduate degree-level nurses required fewer home visits and had better knowledge and health behaviours than those cared for by nurses without such degrees [36]. Again, it is important to keep in mind that current evidence only suggests some trends; it does not offer clear direction on the most effective skill mix for nurses. Those studies that have found positive associations have reported wide-ranging registered nurse proportions: from a low of 46% to a high of 96% [37-39].

A number of studies have examined the added value of specialty certification among physicians. Evidence suggests that physicians with specialty training have lower rates of adverse outcomes for certain procedures and medical conditions. Researchers have found a significant association between greater prior training by physicians on certain surgical procedures and better results in performing those procedures [40-42]. Similarly, patients with acute myocardial infarction tend to have lower risk-adjusted mortality rates when cared for by cardiologists [43]. In pharmacies, meanwhile, the evidence points in the opposite direction. Studies comparing pharmacists to pharmacy technicians have found similar error rates between the two groups [44,45].

#### Balancing junior and senior staff members

This staff-mix conceptualisation draws attention to the proportion of experienced staff members on health care teams. This proportion is usually measured by the number of years an individual has worked in a particular grade or job category. The most common hypothesis is that longer experience is associated with better patient outcomes. However the evidence is scarce and conflicting. Several observational studies have concluded that more years of surgical experience are not associated with lower rates of post-operative complications [46,47]. Similarly, studies suggest no relationship between years of experience as a registered nurse and patient mortality rates [48]. Conversely, others report that for each additional year of nurse experience on a clinical unit there were four to six fewer deaths for every 1000 acute medical patients discharged (depending on hospital type) [49]. Another study demonstrated that registered nurses' duration of practice was inversely related to rates of medication errors and patient falls [50].

#### Mixing disciplines

This conceptualisation involves gathering together individuals from different professions and specialties in order to provide well-rounded care. Multidisciplinary teams are commonly used in hospitals or outpatient services. These primary care teams comprise nurses and physicians, and sometimes include specialists. Collaboration is increasing between mental health and primary care workers, and pharmacists are increasingly integrated into primary care teams [51,52]. Increased interest in a 'whole system' approach to care has also contributed to the inclusion of social service staff, community workers and volunteers on primary care teams [53].

There is an extensive body of literature focusing on the potential benefits of multidisciplinary teams and, more broadly, of collaboration amongst professionals from different disciplines as a way to address fragmentation, discontinuity, and lack of receptiveness. In reality, however, the evidence is inconsistent on the effectiveness of multidisciplinary teams compared to care provided by a single group of professionals. A review of 14 systematic reviews and 33 additional randomised trials found that the impact of multidisciplinary teams on quality of life and clinical outcomes varied considerably amongst the studies [54]. Other research indicates that, although multidisciplinary outpatient teams or teams of primary and secondary care personnel working together can improve patient outcomes; this result may vary according to the initiatives undertaken and patients' conditions. A systematic review focusing on people with rheumatoid arthritis found that multidisciplinary outpatient teams may improve functional outcomes more than usual care [55]. Other trials involving elderly people and those who had suffered strokes, however, found no impact on health outcomes [56,57].

Physician-nurse collaboration has particularly attracted researchers' attention. Some studies suggest that a high degree of collaboration is associated with lower mortality and complication rates and with increased patient satisfaction in adult intensive care units (ICUs) [58,59]. Findings about the value of general practitioner (GP) and nurse collaboration in primary care are often less clear. While some studies have found improved clinical outcomes and satisfaction [60], others have discovered no significant improvement over usual care approaches [61,62].

In addition to the conflicting findings, it is difficult to draw clear conclusions from these studies because most multidisciplinary interventions contain several other variables, such as increased follow-up and medication reviews. It is therefore unclear whether multidisciplinary team composition, additional contacts with staff members, or other factors influence outcomes. Similarly, it is uncertain which specific staff members may be more or less useful within multidisciplinary teams.

#### What can we conclude about optimal staff-mix?

Health care organisations have a range of options for ensuring a richer staff-mix:

• Increasing the number of personnel

• Higher ratios of qualified workers

• Higher ratios of senior staff members

• Multidisciplinary teams

Despite conflicting findings and the need for further research, a number of studies and systematic reviews suggest that a richer staff-mix may be associated with better outcomes and fewer adverse events for patients. The evidence, however, is highly limited by practical limitations and methodological shortcomings. While many studies have reported positive impacts from enriching staff-mix, they do not offer clear guidance about ideal thresholds in terms of personnel/patient ratios or the proportion of different categories of staff members on teams. More fundamentally, the staff-mix perspective that emphasises numbers and types of personnel gives less attention to the conditions that determine how staff members' skills are used. Despite the rhetorical use of 'skill mix' to describe the different options for deploying health care personnel, the focus is, in reality, not on skill but on grades, educational qualifications, job titles and duration of experience that are, at best, proxies for skill levels. An effective system of HR optimisation cannot, however, be restricted to the numbers and types of personnel available. Such a system must also ensure that personnel work to their full potential. Doing so requires a more dynamic approach to skill management that goes beyond the mix of available staff members.

### From staff-mix to skill management

Skill management refers to an organisation's ability to optimise the use of its workforce. The focus shifts here from achieving a specific mix of different types of personnel to adapting workers' attributes - such as knowledge, skills, and behaviours - and roles to changing environmental conditions and demands [63,64]. Skill management enables organisations to optimise patient outcomes while ensuring the most effective, flexible and cost-effective use of human resources. A diverse set of interventions have been tested to achieve this dynamic approach to HR optimisation. We divide them into two main dimensions: skill development and skill flexibility.

#### Skill development

One of the greatest challenges facing health care organisations in recent years has been how to adjust to the rapid pace of a wide variety of internal and external changes:

• Environmental changes in consumers' tastes and demands

• Changes in legal requirements

• Socio-demographic and epidemiologic changes

• Technological developments

• Economic fluctuations.

To a large extent, organisations' strategic and practical adjustments depend on their members' capacity to transform. An organisation updates its responses to changes only when its workforce can learn and utilise the skills required to take on new roles and functions. These additional roles and functions may be at higher, parallel, or even lower level [65], and they can come about through two distinct processes: role enhancement and role enlargement.

##### Role enhancement

Role enhancement involves expanding a group of workers' skills so they can assume a wider and higher range of responsibilities through innovative and non-traditional roles [66]. Enhancing staff members' roles through new competencies gives to employees the opportunity to acquire new competencies and expand their tasks so that they can take on responsibilities traditionally carried out at higher levels [67]. By altering the content of their work, employees are offered opportunities for individual achievement and recognition. Under this model there is greater work depth because employees are involved in tasks that increase their control or responsibility [68]. Role enrichment is considered a vertical and upward expansion of work because it alters authority, responsibility, level of complexity and assignment specificity [69]. In a specific health care context, role enhancement describes a level of practice that maximizes workers' use of in-depth knowledge and skills (related to clinical practice, education, research, professional development, and leadership) to meet clients' health needs [70,71].

Role enhancement does not entail adding functions from other professions. It occurs within a given profession's full scope of practice through the integration of theoretical, research-based and practical knowledge inherent to the development of a discipline [72]. It can also arise from innovative professional activity, new models of health care delivery, and organisational changes that promote development of new knowledge, skills, and practices. Through experience, continued professional growth and development, and collaboration with colleagues from other disciplines, health care workers can develop new skills, abilities, and techniques they did not obtain during previous clinical preparation [73]. In addition, as health care work expands into new settings, the situational factors that shape service provision in those environments create demands for new skills [74].

In health care, role enhancement has been associated with the potential to increase longitudinal and personal continuity and improve patients' health outcomes by enabling one professional to cover a wider range of care needs or by enabling one patient to be cared for by fewer workers. As a result, many health care professionals such as nurses, pharmacists, and GPs have recently expanded their responsibilities beyond their traditional scope of practice to include more innovative roles. In many cases, these role expansions were initiated in order to ensure that individual professionals would be able to oversee a greater proportion of their patients' care.

Primary care and prevention are the main areas in which nurses have taken the lead in delivering expanded services, including health promotion, health screening, and discharge follow-up. Since the 1990s, nurses in UK general practices have been responsible for carrying out well-patient health checks and providing lifestyle counselling and other interventions in accordance with treatment guidelines [75]. Nurses have also expanded their roles by specialising in practice domains and by helping people with particular conditions. Such specialist nurses can be based in either primary or secondary care, and they are particularly active in nurse-led clinics, where nurses assume responsibilities such as managing people with long-term conditions, providing health promotion advice, monitoring and informing patients, and screening for diseases (e.g., cervical screening, cardiovascular screening) [76-79]. Role expansion can also be seen in nurse-led outpatient follow-ups, whereby hospital or community-based nurses oversee discharge planning and post-discharge outpatient follow-up [80]. These examples illustrate the expansion of nursing into areas that were often unmet or inadequately addressed.

While retaining their generalist background, some GPs have also expanded their roles. In the US and the UK, GPs who hold additional qualifications or training and who focus on particular areas are sometimes known as "GPs with special interests." Such physicians can offer specialist care in the community or work as part of multidisciplinary hospital and primary care teams [81-83]. Similar developments have occurred for pharmacists whose work has expanded far beyond the distribution of medications to include patient education, health promotion, counselling, medication management, health monitoring, and even, in some jurisdictions, prescribing [84-86]. In England, the Medicines Management Collaborative involves 146 primary care trusts and 44 trusts, and it aims to engage all members of the pharmacy team in identifying and addressing patients' unmet pharmaceutical needs [87].

Despite major interest in developing enhanced roles, evidence about the impact of these new roles is limited and has focussed mostly on nursing. Overall, the evidence suggests that health professionals can learn specific advanced skills that fall outside the scope of their routine practice and apply them in clinical settings. However the impact of such role enhancement remains uncertain. Some studies have found improvements associated with organisational innovations that draw on nurses with advanced skills, including nurse-led clinics or specialist nurse-led initiatives [88-91]. Other studies have found fewer or no benefits [92-95]. However there are variations in the nursing interventions in these studies which may lead to inconsistencies in the findings and make it difficult to draw conclusions about the effects of enhanced nursing roles on patient outcomes. We cannot be certain whether any observed differences are due to the nurses' roles or to other intervention-related factors (e.g., resource intensity, increased follow-up, access to a multidisciplinary team). Thus, although many studies have revealed connections between nurses' role enhancements and safe and effective care or improved patient outcomes, it remains uncertain whether the benefits are due to specific interventions or nurses' roles. Furthermore, the evidence regarding the opportunity costs of such service developments and marginal gains in terms of health outcomes is still scarce and often conflicting.

In addition to patient outcomes, role enhancement also likely affects professionals. Role enhancement echoes research about motivational theory and job enrichment [96,97]. Motivation may be a function of work factors such as responsibility, advancement, recognition and opportunity to acquire and use vertical skills including, for example, leadership and self-regulation. It has been suggested that enriched jobs that include these factors lead to satisfaction and motivation because they provide workers with more control, responsibility, and discretion over how they perform their jobs. Research on role enhancement in various sectors suggests that enriched jobs are more meaningful and less exhausting and associated with greater job satisfaction [98-101]. In the health care arena, role enhancement may also have a positive effect on workforce recruitment and retention, either by providing more advanced roles with increases in pay and status or through the creation of new clinical career pathways [102].

Despite the benefits associated with role enhancement, some caution is required. First, as traditional roles and functions change, confusion and disagreements can challenge professionals' identities and engender conflicts among practitioners and occupational groups. Such conflicts can, in turn, lead to low morale and antagonistic working relationships [103,104].

Second, work expansion, even in a vertical direction, is not always synonymous with job enrichment or role enhancement. In the absence of an explicit professionalization project, HR management strategies designed to expand practice scopes may undermine professionals' distinctive work domains because they blur role boundaries and make the work of one profession indistinguishable from that of others. Lack of clarity about professional practice means that, in fulfilling useful, flexible, and cost-effective new roles, individuals may serve managerial, economic, and patient interests, but their roles may remain limited and lack any obvious benefits for the development of their professions. Some analysts have even suggested that the skill-mix changes that have recently gained popularity (e.g., addition of new functions to nurses' roles) are nothing more than revamped versions of rationalisation programmes, undertakings that exposed workers to a potent mix of resource constraints, heavy workloads, significant role changes, and pressures to develop a broader range of skills [105,106]. These increased pressures to develop new skills and reach higher educational standards may be counter-productive if they demotivate workers who feel they must take on additional work without reciprocal support [107].

Third, it cannot be assumed that role enhancement means a general upskilling of workers. Just because staff members must perform more tasks at higher levels does not mean they have been supported by further training. Several influential reports have voiced concerns that the broad range of initiatives being implemented to expand health care workers' roles is not always combined with efforts to establish educational and training programs that are consistent with these developments [108,109]. While some key stakeholders, including governments and employers, have argued for the expansion of scopes of practice in health care, the pace of service development has often outstripped the ability of training programs to equip workers.

##### Role enlargement

Role enlargement is the horizontal accrual and diversification of employees' skills. Staff members are able to extend their activities and take on roles and functions at parallel levels (horizontal enlargement) or lower levels (downward enlargement) [110-112].

In industry, role enlargement aims to change the scope of jobs in an attempt to motivate workers [113,114]. This practice emerged as a response to excessive specialisation in the division of industrial labour, whereby work is typically divided into small units, each of which is performed repetitively by an individual worker. Concerns about extreme specialisation and its adverse effects on workers' morale led to calls to restore some of the skill, responsibility, and variety that have been lost through work simplification [115,116].

In health care, role enlargement has been part of efforts to shift service delivery from a task-oriented approach towards integrated care carried out by workers who are able to meet patients' multiple and complex needs [117]. While the rapidly shifting balance between acute and chronic health problems in industrialised countries is placing new demands on health care workers, there is a general consensus that health care professionals' skills must be expanded in order to provide effective care for people with chronic conditions [118]. Population-based approaches to care that have been part of recent reforms in many jurisdictions move health care workers from caring for a single unit (one person seeking care) towards planning and delivering care to defined populations, to ensure that effective interventions reach all the people who need them within a given population. To meet this challenge, practitioners must assume new roles such as the ability to manage populations, to assess the health care needs of wider groups, and to plan and implement appropriate levels of health and social-care interventions.

As with role enhancement, role enlargement succeeds not by replacing one professional with another but by adding new dimensions to health care through the expansion of workers' skill repertoires. Such role enlargement has been present in many recent initiatives in which the main focus has been on practitioners' acquisition of additional, basic patient-care skills. These new skills enable practitioners to perform certain routine, frequently provided, easily trainable, and low-risk procedures (e.g., monitoring vital signs, measuring blood glucose level, carrying out venipuncture for blood sampling, measuring peak expiratory flow rate, examining for breast lumps and providing advice on health promotion) that can help bring about more integrated care.

Horizontal expansion can also be seen in increased interest in cross-training generic and nonclinical skills, such as patient/client education, technical writing and team dynamics/communication. The World Health Organisation (2005) [119] has identified five core generic skills that transcend the boundaries of specific disciplines and apply to everyone who cares for patients with chronic conditions:

• Patient-centred care

• Partnering

• Quality improvement

• Information and communication technology

• A public health perspective.

In addition to completing basic disciplinary training, professionals who care for patients with chronic conditions must acquire a broad range of skills related to programmatic activities, quality improvement, case management, systems design and management of clinical services. In several countries, this role enlargement is reflected in training efforts whereby health care workers learn to negotiate care plans with patients, to support patients' self-management, to use information systems, and to work as members of teams [120].

Beyond its potential to reduce service fragmentation, role enlargement can also have a positive impact on staff members themselves. Studies on the effects of job-enlargement programs have generally shown that focusing on role breadth tends to increase job variety, enhance task significance, increase autonomy, and improve motivation [121-123]. In one study, multi-skilled health care workers with broad practice scopes reported having more interesting jobs, greater job security, and more feelings of enhanced contribution to their hospital than did uni-skilled employees [124].

However some research has also found that role enlargement must be undertaken cautiously because unabated expansion can eventually threaten professional identity, intensify workloads to the point of excess, and spark significant levels of demotivation and dissatisfaction. Nurses, for instance, have reported negative outcomes associated with role enlargement, primarily as a result of having to undertake more tasks. Occurring at a time of nursing shortage and often in the absence of reciprocal workload support from other occupations, these extra demands involve juggling additional functions on top of pre-existing clinical responsibilities and in more pressured environments [125]. In such cases, staff members' resentment is fuelled by the perception that their specialist knowledge and skills are being devalued at the same time as they are being asked to take on a broader range of generic functions while less qualified personnel are taking over their traditional areas of responsibility [125].

#### Skill flexibility

Another closely related dimension of skill management is skill flexibility. This term refers to using multi-skilled workers that can switch from one role to another while employing various skills as required [126]. A multi-skilled workforce capable of doing different jobs and delivering a wide range of services to clients results from increasing the breadth and depth of work. In health care, role substitution and role delegation are two of the main strategies being widely tested.

##### Role substitution

Role substitution involves extending practice scopes by encouraging the workforce to work across and beyond traditional professional divides in order to achieve more efficient workforce deployment [127]. In contrast to role development, which occurs within dynamic disciplinary boundaries, role substitution entails competencies required to perform activities that are usually considered to be outside traditional practice scopes.

In recent decades role substitution has blurred traditional professional boundaries. In the US for example, physician assistants with a wide variety of backgrounds, including nursing and social care, have become an attractive option for expanding workforce capacity in underserved areas [128]. Similarly, in many countries several types of non-professionally qualified staff members have been used as substitutes for nurses. Substitution of less expensive 'care assistants' for more expensive nurses has become increasingly apparent in recent years in response to cost-containment initiatives and nurse shortages. Other role substitution examples include training respiratory therapists to perform electroencephalograms (EEGs) and medical technologists to perform certain radiological procedures [129]. In the field of mental health, nurse practitioners have extended their activities to many areas previously reserved for physicians, including treating depression and anxiety disorders as well as clinically assessing people who are receiving anti-psychotic injections [130-133]. Meanwhile, both family physicians and midwives have been sharing roles with obstetrician/gynaecologists (in prenatal and postnatal care, delivery and routine screening tests).

Over the last few decades, pressures such as rising costs, personnel shortages, and access limitations have raised interest in role substitution as a skill management tool for fostering more cost-effective use of a diversely skilled and flexible workforce [134,135]. But it remains unclear whether role substitution lowers costs.

Substitution of nurses for physicians has received a great deal of research attention. Overall, the evidence supports the view that, in many clinical areas, particularly primary care, there is substantial potential for nurse substitution to lower costs without decreasing quality. Nurses may even extend quality into areas of care not generally provided by physicians [136]. In this respect, several studies have shown that nurses operating in roles that overlap physicians' achieve health outcomes that are as good as those accomplished by physicians and generate higher patient-satisfaction ratings - particularly with regard to interpersonal skills [137-139]. Substituting nurse midwives for physicians has been also well studied and, again, the findings suggest that health outcomes for patients are comparable for both groups, but that midwives may use less technology and analgesia in intrapartum care [140,141].

Substituting less qualified personnel for highly qualified nurses is, however, a contentious practice. Although such role substitution offers a way to cope with staff shortages, many studies have suggested that it may adversely affect patient-related outcomes (e.g., decreased satisfaction, decreased care quality) and nurse-related outcomes (e.g., increased on-call work, increased sick leave and overtime work, increased workload for registered nurses) [142-144].

While workforce substitution is often initiated as a cost-saving strategy, evidence about this is weak. Substitute workers may be able to provide equal quality care, yet the impact on costs depends on a number of factors, including whether substitutes answer previously unmet patient needs or, instead, generate new demands for care. It has been suggested that nurses, compared with physicians, spend more time with patients, recall them at higher rates, and carry out more investigations - all of which have cost implications [145,146]. In addition, although it is generally less expensive to train nurses than physicians, savings may be eroded because nurses tend to have lower lifetime workforce participation rates than doctors. Similarly, while there is no unanimity in this regard, current evidence suggests that substituting nurse aides or nurse assistants for more highly qualified and more expensive nurses may be no more cost-effective because of the various hidden expenses associated with skill dilution: higher absence and turnover rates of less-qualified staff, greater levels of unproductive time due to lack of autonomy and capacity to act independently, and higher rates of adverse events and risks for patients [147,148].

Another danger with role substitution is that skills that are shared by a broad range of professionals may become a low priority for individual practitioners. Increasing the range of people capable of undertaking particular tasks might mean that those tasks are no longer specifically "owned" by anyone. Reports have shown that practices intended to increase continuity have led, in reality, to role and skills drift as well as to more fragmented care [149]. One example is the reduction of medical involvement in maternity care that has occurred in tandem with the extension of midwives' scopes of practice, leading to situations in which physicians no longer see certain tasks (e.g. suturing the perineum after a delivery) as belonging to them.

##### Role delegation

Role delegation involves transferring certain responsibilities or tasks from one grade to another by breaking down traditional job demarcations. In practice, groups of professionals take on roles delegated to them by other groups of professionals. Interest in delegation has been driven by its potential to make highly qualified and high-cost practitioners withdraw from activities that can be competently performed by less qualified and lower-cost practitioners. As a result, the former group can devote more time to the interventions that only they can perform.

Some research suggests that between 25% and 70% of physicians' (most often generalists') tasks could be delegated to other health care professionals [150]. In the same vein, other studies have concluded that GP workload for specific patient groups can be reduced by up to 50% by delegating some activities to nurses, including managing requests for out-of-hours appointments [151], same-day appointments [152], and home visits [153]. A more recent estimate of the Wanless report in the UK is that nurse practitioners could take on about 20% of work currently undertaken by GPs and junior physicians, whilst health care assistants could cover about 12.5% of nurses' current workload [154]. According to other studies, task delegation would allow a significant proportion of nurses' workload to be taken up by health care assistants, auxiliary nurses, and other less-qualified staff members [155,156]. It has been found that in accident and emergency units over a 24-hour period, nursing staff members spent 49% of their time on nursing tasks, 21% on communicating with patients, 17% on clerical work, and 13% on housekeeping. These figures mean that a significant proportion of current nursing work could be delegated to untrained personnel such as health care assistants or support workers.

Evidence concerning the impact of role delegation on both patient and staff outcome is limited and conflicting. The benefits of role delegation need to be balanced by the potential drawbacks that researchers have found. Removing simple tasks from GPs and delegating them to other staff members may affect the sense of connection between patients and their physicians, thus compromising this important relationship [157-159]. Second, removing relatively simple tasks in order to allow physicians and nurses to manage more complex health problems may deprive physicians of valuable interludes in their work and be counterproductive if it leads to increased stress and job dissatisfaction. Furthermore, unless there is a reciprocal helping relationship or additional resources and support, shifting work from higher to lower-skilled groups can lead to excessive workloads for the latter and fuel the perception that one group is off-loading tasks onto another [160,161]. Finally, assessment of the scope for health care role delegation must take account of the context of workforce shortage. If 20% of GPs' and junior physicians' work were shifted to nurses, as suggested by the Wanless report mentioned above, pressure on GPs would decrease. That move could, however, exacerbate nurses' dissatisfaction with their workloads and simply transfer the problem of workforce shortage from one professional group to another.

Role enhancement, role enlargement, role substitution and role delegation are all personnel management tools that divert focus away from the issue of numbers and occupational mix towards the range of roles, functions, responsibilities and activities each staff member is educated and able to perform. These four tactics reflect a more dynamic approach to HR optimisation, one that emphasises responsiveness to patients' needs while enabling providers to practise to the full scope of their abilities. Such an approach is based on the premise that providers' scopes of practice and use of skills may alter over time and across different contexts, whether in response to macro-level system changes (e.g., emphasis on primary health care, shift from institutional to community care, new developments in technology) or evolution at the level of the employment setting (e.g., client needs, organisational resources).

From this perspective, managers are faced with a twofold challenge: creating the conditions so that the human resources at their disposal can develop the skills necessary to fill the new roles imposed by changing services; and finding appropriate mechanisms for ensuring greater flexibility in using the competencies their staff possess. From an instrumental point of view, this implies a stronger emphasis on developing tools that will enable managers to clarify the roles of their staff in different contexts, to monitor the scopes of practice of their staff, and to detect any barrier or facilitator to effective utilisation of the workforce. The managerial and policy challenge is to monitor and narrow the gaps between the potential contribution of health worker (as allowed by the education, knowledge, and skill base) and their actual practice as delimited by legislation, employer policies, experience, and context of practice.

From this perspective, interventions aimed at HR optimisation must target or take account of a range of factors likely to influence scopes of practice and the use of providers' skills:

• Legislation and standards

• Educational programs

• Practice settings (including availability of adequate support systems such as orientation programs and professional development)

• Clients' needs.

The next section outlines some of the organisational and institutional factors needed to optimise HR in health care. These factors are important because they can help managers, practitioners and policy-makers make the best use of available resources, regardless of staff shortages or changing political and organisational contexts.

### Organisational and institutional factors

Limitations in the current evidence on skill mix have been well documented [162]. Studies have been criticised for their methodological flaws, their descriptive focus and their reliance on statistical correlations that fail to account for many key variables [163-165]. In addition, much research was based at single sites, drew on small sample sizes, and was poorly designed - all factors that limit their external validity. Identifying what constitutes appropriate outcomes and linking those outcomes to a particular staffing combination remains contested terrain. Not only are many outcome indicators not easily accessible to researchers, but it is also difficult to determine the specific effects of one staffing mix while controlling for the large number of variables that are likely to influence outcomes [166].

A fundamental reason limiting the conclusions that can be drawn is the lack of a solid theoretical foundation underlying the studies. Much research is based on the premise that some specific HR practices are always better than others and that all organisations should adopt those best practices. One example is the universal nurse ratio promoted in places such as the US and Australia. The evidence for such an approach is based mainly on empirical tests of relationships between one or more independent variables and various dependent variables. Such analyses often show high levels of statistical significance but give no explanation of how human capital was activated. They likewise provide few details of how organisational structures and processes as well as their internal and external environments influence HR practices and outcomes.

**Figure 1:**
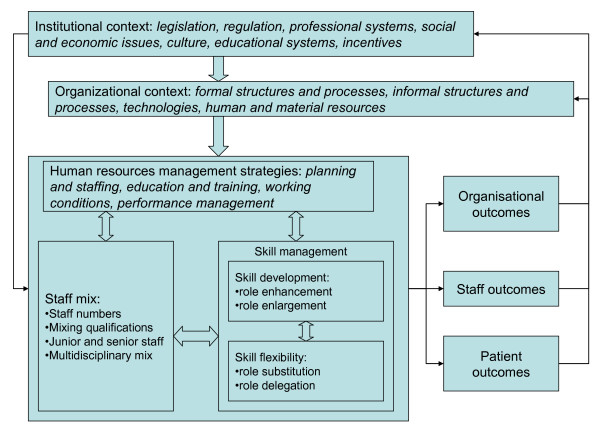
**A framework for optimising human resources in health care**.

Drawing on several decades of empirical research and theoretical developments in the domain of strategic HR management, the framework we propose below (see Figure 1) builds on a system-wide perspective and conceptualises HR optimisation as the result of multiple, integrated, and interacting interventions that concern staff-mix, management of staff members' skills, and practice environments in which personnel apply their skills. The interventions we consider are subject to the influence of both the organisational contexts and the wider environments through which organisations manage their human capital [167-172]. From this system perspective, HR optimisation implies an attempt to achieve a horizontal fit among HR activities and a vertical fit with other organisational policies, goals, and structures, as well externally with the wider operating environment. On the vertical front, HR optimisation depends on congruence between an organisation's strategic context and its staff members' functional practices. Externally, such optimisation depends on the ability to adjust HR practices to the changing sets of rules and requirements imposed upon organisations by their social, legal, and political contexts. In this framework, health care workers respond to the organisations in which they provide care and health care organisations respond to the broader policy environments that influence their personnel.

Although it is important to consider the different levels of determinants that affect health care HR, marking out the boundaries between them is not clear cut. For instance, education and training have long been considered key functions of the HR management sub-system. Yet, organisations are also responsible for identifying and addressing staff members' training deficiencies and for ensuring that providers have the expertise and tools to care for patients. Alternatively, education and training issues can be addressed at the institutional level where decisions are made in order to ensure medical training curricula or continuing education requirements meet population demands.

(Figure 1)

Adopting a systemic view of HR management begins with the recognition that it brings together a number of interdependent functions working in synergy to achieve organisational performance. Analysts suggest that HR management activities are organised around four key functions that can produce different practice configurations [173]:

• Planning and staffing policies

• Education and training resources and structures

• Working conditions

• Performance management.

Because these four functions are interconnected and interactive, HR optimisation depends on a congruent pattern of activities that use them synergistically to develop, organise, manage, and use an organisation's skills stock. HR management systems function best when they all fit with and support each other. For instance, ensuring the availability of an appropriate number of personnel and their adequate distribution will depend on the education system's ability to provide well-trained and competent health care professionals. In contrast, a case in which job structures were based on teams but incentive systems and career opportunities were entirely linked to individual performance would be an example of poor horizontal fit. Overall, the inference is that managing HR in isolation from other organisational functions and systems, or addressing a problem in one function without focusing on interactions with others, are likely to increase the incidence of failure.

#### The organisational context: achieving a vertical fit

Issues of HR development cannot be dealt with in isolation from their organisational contexts. The quality of a service depends on the personnel performing it, but also on the settings in which it develops and on the resources available to provide the service. If transformation in health care organisations is impossible without transformation of the workforces that deliver care, organisational characteristics also frame the possible options for managing available HR. Effective skills management depends not only on the horizontal fit between HR activities but also on the vertical fit between HR practices and organisational contexts.

An organisation is a complex system that builds on its human capital to convert inputs to outputs. This conversion process is achieved through configurations of organisational components consisting of formal and informal structures and processes, cultures and technologies (including procedures, practices, and guidelines). These organisational components provide the day-to-day contexts in which health care workers carry out their tasks. They shape internal structures that govern important staff-related factors, including:

• Number and mix

• Status

• Extent of social contact in the workplace

• Working conditions

• Opportunities for self-development and self-realization.

To the extent that these organisational components are aligned with an organisation's HR needs, a workforce can perform effectively and produce quality outcomes.

There is no single, most appropriate organisational structure or process for optimising personnel performance. However, the extent to which any organisational structure or process is able to stimulate workers' performance depends on how well its components are articulated and facilitate staff members' ability to meet organisational goals.

Several organisational characteristics appear to determine which HR variables affect patient outcomes. Findings from studies on magnet hospitals, for instance, indicate that key patient outcomes, as well as health care workers' improved work-related well-being, depend on the organisational characteristics that create conditions for professionally based practice environments [174,175]. Those organisation-level elements include:

• Relatively flat hierarchy with few supervisors

• Worker autonomy

• Participative management

• Professional development opportunities

• A relatively high organisational status for nursing

• Collaboration

Research into high-performing workplaces also suggests that positive outcomes can be produced through cumulative and synergistic effects among reinforcing 'bundles' of organisational practices. These findings have emerged from four main research streams. The first is the teamwork perspective. Research in this area suggests that organisation-level factors that support teamwork such as organisational structures, management/strategies, and resources/tools strongly influence both the development of health care teams' collaborative practices as well as their outcomes [176-180].

The second research stream is the high-involvement perspective. From this perspective, organisational characteristics that foster empowerment, decision ownership, job autonomy/discretion and participation boost workers' productivity by engaging them in a more responsible and a more responsive manner [181-183].

The third research stream explicitly examines the connections between organisational social climate and employee performance. Experts postulate that features that define organisational social climate affect personal attitudes and behaviours and, as a result, organisational performance [184-186]. Empirical studies have reinforced these hypotheses. For example, researchers have demonstrated that a climate high in autonomy and supportiveness is positively related to job performance [187,188]. Health care workers may also be more motivated to perform well if their organisations and managers were to provide a clear sense of vision and mission, increase staff members' participation in decision-making, encourage teamwork, foster innovation, provide career structures and opportunities for promotion, and use available sanctions for poor performance in ways that are fair and consistent [189-191].

HR interventions must be aligned with culture at the organisational level. Managerial style, evaluation and reward systems, accountability, decision latitude, and vehicles and opportunities for employee feedback all reflect an organisation's culture. Evidence suggests that all these factors may influence an individual worker's level of commitment and motivation, and, therefore, levels of skills retention, skills utilisation, and skills development across an entire workforce [192-195].

In Western societies, health care organisations operate in environments characterised by continual developments in the content of services and the technologies used to deliver them. Making the best use of health care providers therefore depends upon the availability of requisite technologies (including procedures, guidelines protocols, and medications) and their appropriate utilisation. In this respect, a growing amount of evidence suggests that the automation of clinical, financial, and administrative transactions allowed by new information technologies can lead to health care workforce productivity gains [196,197]. These arise as a result of improving the ways staff members provide clinical and public health care services and by reducing the cost of service provision and, hence, freeing up resources to provide care for other patients. In a similar vein, Shortell et al. [198] studied the role of organisational factors in determining the performance of hospital ICUs. The authors found that the availability of state-of-the-art technology was a statistically significant determinant of risk-adjusted patient mortality.

These examples illustrate how organisational structures, processes, and technologies offer many levers for optimising health care HR. Misalignment among these organisational components and an HR sub-system may result in sub-optimisation of an available workforce. Interventions designed to improve workers' performance should not be restricted to one of these organisational components. Rather, a combination of interventions cutting across organisational components is more likely to form an internally consistent and reinforcing work environment. Such an approach also moves HR management activities from an operational and technical level to a more strategic one, where the focus is not only on developing a set of coherent workforce policies and practices but also on ensuring that employees' collective knowledge, skills, and abilities contribute to achieving organisational objectives. This strategic approach creates conditions that favour win-win scenarios that improve organisations' prospects of achieving their outcomes while benefitting employees through better work practices [199-202].

#### The institutional context: achieving an external fit

Optimising health care personnel must also consider institutional context. Health care delivery occurs in highly institutionalised environments, settings that differentiate it from other human service systems. The regulations that govern health care organisations and workers are extremely dense and diverse. The institutions and agencies involved in these processes are also pluralistic, requiring the development of complex linkages among various bodies. Components of the institutional environment include:

• Political structures that define the distribution of responsibilities and power between various occupational groups

• Rules, regulations, and laws that govern provider behaviour and working conditions

• Regulatory bodies that assume control of professional activities

• Policies and legislation that provide incentives to health care professionals to improve their practice

Together, these components create the broad social, cultural, economic, professional, and political context in which key HR decisions are made.

Issues related to institutional context may both enable and constrain personnel optimisation. More flexible use of workers is often considered an important tool for making health care more responsive to consumers' needs; however, this has often been difficult to accomplish due to regulatory constraints. International variations in the scope of practice of health care professionals suggest that groupings of skills into professions are often arbitrary and owe more to custom, traditions, incentives, professional politics, and power than to logic and providers' actual skills [203,204]. A number of reports have highlighted that entrenchment of scope-of-practice rules and outdated legislation have resulted in inefficient use of scarce HR in many areas [205,206]. In some cases, rules prevent health care professionals from providing the full range of services they have been trained to deliver. In other cases, lack of a coherent regulatory framework creates obstacles to delivery. One often-cited example is that of nurse practitioners, who in many countries remain constrained by the medical profession's scope-of-practice rules.

In many sectors, technological advances have resulted in increased productivity and lower cost-per-unit of service; that result has been less obvious in health care. Highly educated and skilled professionals must spend inordinate amounts of time on matters that could be handled by other staff members. Registered nurses, for instance, are often restricted from tasks for which they are fully qualified and are directed because they have to perform non-nursing duties such as answering telephones, collecting meal trays, and scrubbing bathtubs [207]. This type of personnel deployment is costly and makes for less-satisfying work for qualified professionals. Other examples might be drawn from technological developments in the area of surgery. The dramatic increase in productivity - a development that has been observed in for several interventions, such as cataract and arthroscopic surgery - has often resulted in higher incomes for practitioners but not in lower costs for taxpayers.

Occupational regulations and scope-of-practice rules are just one aspect within a complex regulatory system that encompasses the educational and incentives systems. Over the last decade many governments have introduced health care reforms with the promise of better utilising the spectrum of health care providers through inter-professional teamwork and integration of health care services. In contrast to rhetorical claims for inter-professional teamwork, however, the educational preparation of health care workers remains relatively entrenched in the traditional paradigm where opportunities for interdisciplinary learning that prepare formal caregivers to work cooperatively across professional boundaries have been limited [208]. For the most part, health care professionals continue to be trained in separate compartments, with little shared training in areas of common concern and few opportunities to develop skills and competencies to enable them to function in teams [209].

The optimal use and effective management of skills depends on the incentives built into a health care system. Financial incentives play a major role in defining professionals' roles and in whether health care providers embrace or resist changes in their mix of skills and responsibilities. One often-cited example is the comparison of scopes of practice of obstetrics and anaesthetics in the US and UK. Routine childbirth is managed by midwives in the UK, while in the US midwifery by qualified nurses has been slow to develop and many babies are still delivered by obstetricians. In contrast, nurses in the US often administer routine anaesthesia, but in the UK that procedure is the preserve of physicians (although attempts have been made recently to replicate the system of nurse anaesthetists in the UK) [210]. One suggested explanation for these variations between the US and the UK is the difference in the payment system of providers [211]. In the US fee-for-service system, dominant physicians have no incentive to share with midwives the lucrative baby-delivery market. However, when they are performing surgeries there is a stronger incentive for physicians to split their fees with nurse anaesthetists than with more expensive physicians. Such incentives are absent in the UK's salaried approach to physician compensation.

More generally, health care services' funding mechanisms have been consistently identified as either facilitating or blocking the optimal use of providers. When physicians rely primarily on fee-for-service compensation, expanding the role of other professionals in team-based care may be seen as taking away physicians' income. In contrast, in practice settings in which teams rather than individuals are funded, teams would be more likely to look for ways to optimize the use of their different staff members. The UK system of paying the practice (not individual providers), the Australian experience of promoting integrated health care teams, and the innovative reimbursement models instituted through the intergovernmental Primary Health Care Transition Fund in Canada illustrate how compensation reform can help to improve personnel utilisation [212,213].

Developing new roles and searching for more flexibility in using staff members requires an assessment of the environmental conditions that influence health care workers' practices. In order to use limited HR more effectively, it is also necessary to change certain institutional and legal systems in order to accomplish the following:

• Alter the incentives for the various health care professions

• Enhance collaboration and multidisciplinary approaches

• Facilitate work across professional divides

• Ensure that the most appropriately qualified health care personnel deliver the requisite care

## Conclusion

This article has summarised different approaches to optimising HR in health care. We have argued that perspectives that focus on staff-mix, such as those that count the number of personnel needed or focus on generating formulae and algorithms, provide only partial solutions. Wider perspectives, which focus on how human resources can be differently managed either through skill development or skill flexibility, go some way towards conceptualising personnel use in the dynamic and constantly evolving realm of health care. In order to be fully effective, policy-makers, managers, and practitioners need to consider the organisational factors that affect how staff members work. The evidence suggests that no matter which workers are employed or what their roles are, it is only by tackling organisational issues that a fully efficient and effective workforce can be generated. In order to use human resources most effectively, organisations must also consider the institutional environments that frame health care workers' educational preparation, the system of professional regulation, organisational incentives, and the broad range of levers that can be mobilised at both organisational and system levels.

## Competing interests

The authors declare that they have no competing interests.

## Authors' contributions

CAD contributed to methods, literature search, and wrote successive drafts of the manuscript. DS contributed to methods, literature search, articles screening and wrote and reviewed successive drafts.

## References

[B1] ACHDHR A framework for collaborative Pan-Canadian health human resources planning 2007 Ottawa: Health Canada, Advisory Committee on Health Delivery and Human Resources, HHR Planning Subcommittee

[B2] Dussault G Dubois C-A Human resources for health policies: a critical component in health policies Human Resources for Health 2003 1 1 10.1186/1478-4491-1-1 12904254 PMC166115

[B3] Joint Learning Initiative Human resources for health: overcoming the crisis 2004 Cambridge: Harvard University Press

[B4] World Health Organization Working together for health - the World Health Report 2006. Geneva 2006

[B5] Buerhaus P Staiger D Auerbach D The future of the nursing workforce in the United States. Data, trends, and implications 2008 Sudbury, MA: Jones and Bartlett Publishers

[B6] Cooper RA Getzen TE McKee HJ Laud P Economic and demographic trends signal an impending physician shortage Health Affairs 2002 21 1 140 154 10.1377/hlthaff.21.1.140 11900066

[B7] Diallo K Zurn P Gupta N Dal Poz M Monitoring and evaluation of human resources for health: an international perspective Human Resources for Health 2003 1 3 10.1186/1478-4491-1-3 12904252 PMC179874

[B8] Romanow RJ Building on values: the future of health care in Canada 2002 Saskatoon: Commission on the Future of Health Care in Canada

[B9] Organization for Economic Cooperation and Development Towards high-performing health systems 2004 Paris: OECD

[B10] Dixon-Woods M Cavers D Agarwal S Annandale E Arthur A Harvey J Hsu R Katbamna S Olsen R Smith L Riley R Sutton AJ Conducting a critical interpretive synthesis of the literature on access to healthcare by vulnerable groups BMC Med Res Methodol 2006 6 35 10.1186/1471-2288-6-35 16872487 PMC1559637

[B11] Bloor K Maynard A Planning human resources in health care: towards an economic approach 2003 Ottawa: Canadian Health Services Research Foundation

[B12] Duffield C Forbes J Fallon A Roche M Wise W Merrick ET Nursing skill mix and nursing time: the roles of registered nurses and clinical nurse specialists Aust J Adv Nurs 2005 23 2 14 21 16502964

[B13] Buchan J Dal Poz MR Skill mix in the health care workforce: reviewing the evidence Bull World Health Organ 2002 80 7 575 80 12163922 PMC2567564

[B14] Buchan J Ball J O'May F Determining skill mix in the health workforce: guidelines for managers and health professionals 2000 Geneva: World Health Organization

[B15] Hassell K Shann P Noyce P The complexities of skill-mix in community pharmacy Pharmaceutical Journal 2002 269 851 854

[B16] Carr-Hill R Currie L Dixon P Skill mix in secondary care: SDO scoping exercise: Final report 2003 UK: University of York Centre for Health Economics

[B17] Tourangeau AE Cranley LA Jeffs L Impact of nursing on hospital patient mortality: a focused review and related policy implications Quality and Safety in Health Care 2006 15 1 4 8 10.1136/qshc.2005.014514 16456202 PMC2563988

[B18] Unruh L Licensed nurse staffing and adverse outcomes in hospitals Med Care 2003 41 1 142 52 10.1097/00005650-200301000-00016 12544551

[B19] McGillis Hall L Doran D Baker GR Pink GH Sidani S O'Brien-Pallas L Donner GJ Nurse staffing models as predictors of patient outcomes Medical Care 2003 41 9 1096 1109 10.1097/01.MLR.0000084180.07121.2B 12972849

[B20] McGillis Hall L Doran D Pink GH Nursing staffing mix models, nursing hours and patient safety outcomes JONA 2004 34 1 41 45 10.1097/00005110-200401000-00009 14737034

[B21] Needleman J Buerhaus P Mattke S Stewart M Zelevinsky K Nurse staffing levels and the quality of care in hospitals N Engl J Med 2002 346 22 1715 22 10.1056/NEJMsa012247 12037152

[B22] Person SD Allison JJ Kiefe CI Weaver MT Williams OD Centor RM Weissman NW Nurse staffing and mortality for medicare patients with acute myocardial infarction Medical Care 2004 42 1 4 12 10.1097/01.mlr.0000102369.67404.b0 14713734

[B23] Rogers AE Hwang WT Scott LD Aiken LH Dinges DF The working hours of hospital staff nurses and patient safety Health Affairs 2004 23 4 202 212 10.1377/hlthaff.23.4.202 15318582

[B24] Aiken LH Clarke SP Sloane DM Sochalski J Silber JH Hospital nurse staffing and patient mortality, nurse burnout, and job dissatisfaction JAMA 2002 288 16 1987 93 10.1001/jama.288.16.1987 12387650

[B25] Hickam DH Severance S Feldstein A Ray L Gorman P Schuldheis S The Effect of Health Care Working Conditions on Patient Safety. Evidence Report/Technology Assessment, Number 74 2003 Rockville, MD: Agency for Healthcare Research and Quality PMC4781355 12723164

[B26] Nash IS Corrato RR Dlutowski MJ O'Connor JP Nash DB Generalist versus specialist care for acute myocardial infarction American Journal of Cardiology 1999 83 5 650 654 10.1016/S0002-9149(98)00961-8 10080413

[B27] Ellis SG Weintraub W Holmes D Shaw R Block PC King SB III Relation of operator volume and experience to procedural outcome of percutaneous coronary revascularization at hospitals with high interventional volumes Circulation 1997 95 11 2479 84 9184577 10.1161/01.cir.95.11.2479

[B28] Hartz AJ Kuhn EM Pulido J Prestige of training programs and experience of bypass surgeons as factors in adjusted patient mortality rates Medical Care 1999 37 1 93 103 10.1097/00005650-199901000-00013 10413397

[B29] Halm EA Lee C Chassin M How is volume related to quality in health care? A systematic review of the literature 2000 Washington DC: Institute of Medicine 10.7326/0003-4819-137-6-200209170-00012 12230353

[B30] Malenka DJ McGrath PD Wennberg DE Ryan TJ Jr Kellett MA Jr Shubrooks SJ Jr Bradley WA Hettlemen BD Robb JF The relationship between operator volume and outcomes after percutaneous coronary interventions in high volume hospitals in 1994-1996: the northern New England experience. Northern New England Cardiovascular Disease Study Group J Am Coll Cardiol 1999 34 5 1471 80 10.1016/S0735-1097(99)00393-9 10551694

[B31] Klein LW Schaer GL Calvin JE Palvas B Allen J Loew J Uretz E Parrillo JE Does low individual operator coronary interventional procedural volume correlate with worse institutional procedural outcome? J Am Coll Cardiol 1997 30 4 870 7 10.1016/S0735-1097(97)00272-6 9316511

[B32] Khuri SF Daley J Henderson W Hur K Hossain M Soybel D Kizer KW Aust JB Bell RH Jr Chong V Demakis J Fabri PJ Gibbs JO Grover F Relation of surgical volume to outcome in eight common operations. Results from the VA National Surgical Quality Improvement Program Ann Surg 1999 230 414 432 10.1097/00000658-199909000-00014 10493488 PMC1420886

[B33] Hannan EL Siu AL Kumar D Kilburn H Jr Chassin MR The decline in coronary artery bypass graft surgery mortality in New York State. The role of surgeon volume JAMA 1995 273 3 209 13 10.1001/jama.273.3.209 7807659

[B34] Blegen MA Vaughn TE Goode CJ Nurse experience and education: effect on quality of care J Nurs Adm 2001 31 1 33 9 10.1097/00005110-200101000-00007 11198839

[B35] Aiken L Clarke S Cheung R Sloane D Silber J Educational levels of hospital nurses and surgical patient. mortality JAMA 2003 290 12 1617 1623 10.1001/jama.290.12.1617 14506121 PMC3077115

[B36] O'Brien-Pallas L Doran DI Murray M Cockerill R Sidani S Laurie-Shaw B Lochhass-Gerlach J Evaluation of a clientcare delivery model, part 2: Variability in client outcomes incommunity home nursing Nursing Economic$ 2002 20 1 13 21 36 11892543

[B37] Blegen M Goode C Reed L Nurse staffing and patient outcomes Nursing Research 1998 47 43 50 10.1097/00006199-199801000-00008 9478183

[B38] Cho SH Ketefian S Barkauskas VH Smith DG The effects of nurse staffing on adverse events, morbidity, mortality and medical costs Nurs Res 2003 52 2 71 9 10.1097/00006199-200303000-00003 12657982

[B39] Needleman J Buerhaus PI Mattke S Stewart M Zelevinsky K Nurse staffing and patient outcomes in hospitals. Final report 2001 Washington, DC: U.S. Department of Health and Human Services

[B40] Dorrance HR Docherty GM O'Dwyer PJ Effect of surgeon specialty interest on patient outcome after potentially curative colorectal cancer surgery Dis Colon Rectum 2000 43 4 492 8 10.1007/BF02237192 10789744

[B41] Fleischer AB Jr Feldman SR Barlow JO Zheng B Hahn H Chuang TY Draft KS Golitz LE Wu E Katz AS Maize JC Knapp T Leshin B The specialty of the treating physician affects the likelihood of tumor-free resection margins for basal cell carcinoma: results from a multi-institutional retrospective study J Am Acad Dermatol 2001 44 2 224 30 10.1067/mjd.2001.110396 11174379

[B42] Gerbert B Maurer T Berger T Pantilat S McPhee SJ Wolff M Bronstone A Caspers N Primary care physicians as gatekeepers in managed care. Primary care physicians' and dermatologists' skills at secondary prevention of skin cancer Arch Dermatol 1996 132 9 1030 8 10.1001/archderm.132.9.1030 8795541

[B43] Nash IS Nash DB Fuster V Do cardiologists do it better? J Am Coll Cardiol 1997 29 3 475 8 10.1016/S0735-1097(96)00528-1 9060880

[B44] Ness JE Sullivan SD Stergachis A Accuracy of technicians and pharmacists in identifying dispensing errors Am J Hosp Pharm 1994 51 354 357 8160687

[B45] McGhan WF Smith WE Adams DW A randomized trial comparing pharmacists and technicians as dispensers of prescriptions for ambulatory patients Med Care 1983 21 4 445 53 10.1097/00005650-198304000-00007 6843197

[B46] Goodwin AT Birdi I Ramesh TP Taylor GJ Nashef SA Dunning JJ Large SR Effect of surgical training on outcome and hospital costs in coronary surgery Heart 2001 85 4 454 7 10.1136/heart.85.4.454 11250976 PMC1729696

[B47] Eldar S Kunin J Chouri H Sabo E Matter I Nash E Schein M Safety of laparoscopic cholecystectomy on a teaching service: a prospective trial Surg Laparosc Endosc Percut Tech 1996 6 3 218 20 10.1097/00019509-199606000-00011 8743367

[B48] Sasichay-Akkadechanunt T Scalzi CC Jawad AF The Relationship between nurse staffing and patient outcomes Journal of Nursing Administration 2003 33 9 478 85 10.1097/00005110-200309000-00008 14501564

[B49] Tourangeau AE Giovanetti P Tu JV Wood M Nursing-related determinants of 30-day mortality for hospitalized patients Canadian Journal of Nursing Research 2002 33 4 71 88 11998198

[B50] Blegen MA Vaughn TE Goode CJ Nurse experience and education: effect on quality of care J Nurs Adm 2001 31 1 33 9 10.1097/00005110-200101000-00007 11198839

[B51] Singh D Which staff improve care for people with long term conditions. A rapid review of the literature 2005 University of Birmingham, Health Services Management Centre

[B52] Wagner EH The role of patient care teams in chronic disease management BMJ 2000 320 7234 569 72 10.1136/bmj.320.7234.569 10688568 PMC1117605

[B53] Sommers LS Marton KI Barbaccia JC Randolph J Physician, nurse, and social worker collaboration in primary care for chronically ill seniors Arch Intern Med 2000 160 1825 1833 10.1001/archinte.160.12.1825 10871977

[B54] Singh D Which staff improve care for people with long term conditions. A rapid review of the literature 2005 University of Birmingham, Health Services Management Centre

[B55] Vliet Vlieland TP Hazes JM Efficacy of multidisciplinary team care programs in rheumatoid arthritis Sem Arthritis Rheum 1997 27 2 110 22 10.1016/S0049-0172(97)80011-X 9355209

[B56] Caplan GA Williams AJ Daly B Abraham K A randomized, controlled trial of comprehensive geriatric assessment and multidisciplinary intervention after discharge of elderly from the emergency department - the DEED II study J Am Geriatr Soc 2004 52 9 1417 23 10.1111/j.1532-5415.2004.52401.x 15341540

[B57] Donnelly M Power M Russell M Fullerton K Randomized controlled trial of an early discharge rehabilitation service: the Belfast Community Stroke Trial Stroke 2004 35 1 127 33 10.1161/01.STR.0000106911.96026.8F 14671238

[B58] Mitchell RH Armstrong S Simpson TE Lentz M American association of critical-care nurses demonstration projects: Profile of excellence in critical care nursing Heart & Lung: The Journal of Critical Care 1989 18 3 219 237 2722533

[B59] Knaus WA Draper EA Wagner DP Zimmerman JE An evaluation of outcome from intensive care in major medical centers Annals of Internal Medicine 1986 104 3 410 418 3946981 10.7326/0003-4819-104-3-410

[B60] Lozano P Finkelstein JA Carey VJ Wagner EH Inui TS Fuhlbrigge AL Soumerai SB Sullivan SD Weiss ST Weiss KB A multisite randomized trial of the effects of physician education and organizational change in chronic-asthma care: health outcomes of the Pediatric Asthma Care Patient Outcomes Research Team II Study Arch Pediatr Adolesc Med 2004 158 9 875 83 10.1001/archpedi.158.9.875 15351753

[B61] Taylor KI Oberle KM Crutcher RA Norton PG Promoting health in type 2 diabetes: nurse-physician collaboration in primary care Biol Res Nurs 2005 6 3 207 15 10.1177/1099800404272223 15583361

[B62] Aigner MJ Drew S Phipps J A comparative study of nursing home resident outcomes between care provided by nurse practitioners/physicians versus physicians only J Am Med Dir Assoc 2004 5 1 16 23 14706124

[B63] Wright PM Dunford BB Snell SA Human resources and the resource-based view of the firm Journal of Management 2001 27 701 721 10.1177/014920630102700607

[B64] Wright PM Snell SA Toward a unifying framework for exploring fit and flexibility in strategic human resource management Academy of Management Review 1998 23 756 772 10.2307/259061

[B65] Bamberg R Blayney KD Vaughn DG Wilson BR Multiskilled health practitioner education: A national perspective 1989 Birmingham, AL: University of Alabama at Birmingham, School of Health Related Professions

[B66] Sibbald B Shen J McBride A Changing the skill-mix of the health care workforce Journal of Health Services Research and Policy 2004 9 suppl 1 28 38 10.1258/135581904322724112 15006226

[B67] Garg P Rastogi R New model of job design: motivating employees' performance Journal of Management Development 2006 25 6 572 587 10.1108/02621710610670137

[B68] Cook CW Hunsaker PL Coffey RE Management and organizational behaviour 1997 Boston: Irvin McGraw-Hill

[B69] Oztürk H Bahcecik N Baumann SL Nursing satisfaction and job enrichment in Turkey Nursing Science Quarterly 2006 19 360 365 10.1177/0894318406293122 16982725

[B70] Ackerman MH Norsen L Martin B Wiedrich J Kitzman HJ Development of a model of advanced practice American Journal of Critical Care 1996 5 68 73 8680496

[B71] Moloney-Harmon PA The synergy model in practice Critical Care Nurse 1999 19 101 104 10401307

[B72] Bryant-Lukosius D Dicenso A Browne G Pinelli J Advanced practice nursing roles: development, implementation and evaluation The Journal of Advanced Nursing 2004 48 5 519 529 10.1111/j.1365-2648.2004.03234.x 15533090

[B73] American Speech-Language-Hearing Association Position statement: Multiskilled personnel Asha 1997 39 Suppl 17 Spring 13

[B74] Vanier C Hébert M An occupational therapy course on community practice Can J Occup Ther 1995 62 2 76 81 10143441 10.1177/000841749506200205

[B75] Atkin K Hirst M Lunt N Parker G The role and self-perceived training needs of nurses employed in general practice: observations from a national census of practice nurses in England and Wales J Adv Nurs 1994 20 46 52 10.1046/j.1365-2648.1994.20010046.x 7930126

[B76] Brown K Williams EI Groom L Health checks on patients 75 years and over in Nottinghamshire after the new GP contract British Medical Journal 1992 305 629 631 10.1136/bmj.305.6854.619 PMC1883333 1393076

[B77] Calnan M Cant S Williams S Killoran A Involvement of the primary health care team in coronary heart disease prevention British Medical Journal 1994 44 224 228 PMC1238871 8204337

[B78] Muir J Lancaster T Jones L Yudkin P Effectiveness of health checks conducted by nurses in primary care. Final results of the OXCHECK study British Medical Journal 1995 310 1099 104 7742676 PMC2549499

[B79] Tulloch A Screening elderly patients Practitioner 1992 1520 1022 1026 1305746

[B80] Singh D Transforming chronic care: evidence about improving care for people with long-term conditions 2005 Birmingham: University of Birmingham Health Services Management Centre

[B81] Royal College of General Practitioner General Practitioners with Specialist Interests 2004 London: RCGP, Information leaflet 11

[B82] Woodroffe E Nurse-led general practice: the changing face of general practice Br J Gen Pract 2006 56 529 632 633 16882391 PMC1874537

[B83] Wright AF GP 2000: a general practitioner for the new millennium Br J Gen Pract 1996 46 4 5 8745843 PMC1239502

[B84] Chapman JL Zechel A Carter YH Abbott S Systematic review of recent innovations in service provision to improve access to primary care Br J Gen Pract 2004 54 374 381 15113523 PMC1266174

[B85] Iversen L Mollison J MacLeod TN Attitudes of the general public to the expanding role of community pharmacists: a pilot study Fam Pract 2001 18 5 534 6 10.1093/fampra/18.5.534 11604378

[B86] Royal Pharmaceutical Society of Great Britain Pharmacy in a new age: building the future 1997 London: Royal Pharmaceutical Society of Great Britain

[B87] Department of Health Management of medicines - a resource to support implementation of the wider aspects of medicines management for the National Service Frameworks for diabetes renal services and long-term conditions 2004 London: Department of Health

[B88] New JP Mason JM Freemantle N Teasdale S Wong LM Bruce NJ Burns JA Gibson JM Specialist nurse-led intervention to treat and control hypertension and hyperlipidemia in diabetes (SPLINT): a randomized controlled trial Diabetes Care 2003 26 8 2250 5 10.2337/diacare.26.8.2250 12882844

[B89] Vrijhoef HJ Diederiks JP Spreeuwenberg C Wolffenbuttel BH Substitution model with central role for nurse specialist is justified in the care for stable type 2 diabetic outpatients J Adv Nurs 2001 36 546 55 10.1046/j.1365-2648.2001.02007.x 11703549

[B90] Connor CA Wright CC Fegan CD The safety and effectiveness of a nurse-led anticoagulant service J Adv Nurs 2002 38 407 15 10.1046/j.1365-2648.2002.02198.x 11985692

[B91] Ryden MB Snyder M Gross CR Savik K Pearson V Krichbaum K Mueller C Value-added outcomes: the use of advanced practice nurses in long-term care facilities Gerontologist 2000 40 6 654 62 11131082 10.1093/geront/40.6.654

[B92] Smith B Appleton S Adams R Southcott A Ruffin R Home care by outreach nursing for chronic obstructive pulmonary disease Cochrane Database Syst Rev 2001 3 CD000994 11686972 10.1002/14651858.CD000994

[B93] Salisbury C Francis C Rogers C Parry K Thomas H Chadwick S Turton P A randomised controlled trial of clinics in secondary schools for adolescents with asthma Br J Gen Pract 2002 52 485 988 996 12528584 PMC1314468

[B94] Loveman E Royle P Waugh N Specialist nurses in diabetes mellitus Cochrane Database Syst Rev 2003 2 CD003286 12804458 10.1002/14651858.CD003286 PMC8407322

[B95] Gradwell C Thomas KS English JS Williams HC A randomized controlled trial of nurse follow-up clinics: do they help patients and do they free up consultants' time? Br J Dermatol 2002 147 3 513 7 10.1046/j.1365-2133.2002.04901.x 12207593

[B96] Herzberg F Mausner B Snyderman B The motivation to work 1959 New York: John Wiley

[B97] Hackman J Oldham G Development of the Job Diagnostic Survey Journal of Applied Psychology 1975 60 2 159 170 10.1037/h0076546

[B98] Newman GA Edwards JE Raju NS Organizational development interventions: A meta analysis of their effects on satisfaction and other attitudes Personnel Psychology 1989 42 461 483 10.1111/j.1744-6570.1989.tb00665.x

[B99] Cherniss C Professional Burnout in Human Service Organizations 1980 New York: Praeger

[B100] Farber BA Crisis in Education: Stress and Burnout in the American Teachers 1991 San Francisco: Jossey-Bass

[B101] Kivimäki M Voutilainen P Koskinen P Job enrichment, work motivation, and job satisfaction in hospital wards: testing the job characteristics model J Nurs Manag 1995 3 2 87 91 10.1111/j.1365-2834.1995.tb00086.x 7735655

[B102] Collins K Jones ML McDonnell A Read S Jones R Cameron A Do new roles contribute to job satisfaction and retention of staff in nursing and professions allied to medicine J Nurs Manag 2000 8 1 3 12 10.1046/j.1365-2834.2000.00149.x 11013536

[B103] Dickson N Pearson P Emmerson P Davison N Griffith M Are nurse practitioners merely substitute doctors Prof Nurse 1996 11 325 328 8604430

[B104] Wilson A Pearson D Hassey A Barriers to developing the nurse practitioner role in primary care - the GP perspective Fam Pract 2002 19 641 646 10.1093/fampra/19.6.641 12429668

[B105] Hanlon G Professionalism as enterprise: service class politics and the redefinition of professionalism Sociology 1998 32 43 63 10.1177/0038038598032001004

[B106] Furlong S Glover D Confusion surrounds piecemeal changes in nurses' roles. Nurs Times 1998 94 37 54 55 9832790

[B107] Buchan J Edwards N Nursing numbers in Britain: the argument for workforce planning BMJ 2000 320 7241 1067 70 10.1136/bmj.320.7241.1067 10764372 PMC1117947

[B108] World Health Organization Innovative care for chronic conditions: Building blocks for action 2002 Geneva: World Health Organization

[B109] World Health Organization Preparing a health care workforce for the 21st century: the challenge of chronic conditions 2005 Geneva: World Health Organization

[B110] Oztürk H Bahcecik N Baumann SL Nursing satisfaction and job enrichment in Turkey Nursing Science Quarterly 2006 19 360 365 10.1177/0894318406293122 16982725

[B111] O'Reilly J Where do you draw the line? functional flexibility, training & skill in Britain & France Work, Employment & Society 1992 6 3 369 396

[B112] Buhler PM Are you getting the most out of your employees Supervision 1990 51 10 14 16

[B113] Walker CR The Problem of the repetitive job Harvard Business Review 1950 28 3 54 58

[B114] Elliott JD Increasing office productivity through job enlargement The human side of the office manager's job 1953 New York: American Management Association, Office Management Series, number 134 1 13

[B115] Guest RH Job enlargement - a revolution in job design Personnel Administration 1957 20 2 13 14

[B116] Conant E Kilbridge M An interdisciplinary analysis of job enlargement: technology, costs, and behavioural implications Industrial and Labour Relations Review 1965 18 377 395 10.2307/2520910

[B117] Dubois C-A Singh D Jiwani I Nolte E The human resource challenge in chronic care Caring for people with chronic conditions - a health system perspective 2008 McKee MBerkshire/New York: Open University Press/McGraw-Hill Education, chapter in press

[B118] Duckett SJ Interventions to facilitate health workforce restructure Aust New Zealand Health Policy 2005 29 2 14 10.1186/1743-8462-2-14 PMC1200557 15987520

[B119] World Health Organization Preparing a health care workforce for the 21st century: the challenge of chronic conditions 2005 Geneva: World Health Organization

[B120] Pruitt SD Epping-Jordan JE Preparing the 21st century global healthcare workforce BMJ 2005 330 637 639 10.1136/bmj.330.7492.637 15774994 PMC554912

[B121] Jenkins GD Jr Gupta N The payoffs of paying for knowledge National Productivity Review 1985 4 2 121 130 10.1002/npr.4040040203

[B122] Knouse SB Variations on skill-based pay for total quality management SAM AdvancedManagement Journal 1995 60 1 34 38

[B123] Wong CS Campion MA Development and test of a task-level model of motivational job design Journal of Applied Psychology 1991 76 825 837 10.1037/0021-9010.76.6.825

[B124] Vaughan DD Fottler MD Bamberg R Blayney KD Utilization and management of multiskilled health practitioners in US hospitals Hospital and Health Services Administration 1991 36 3 397 419 10112576

[B125] Adams A Lugsden E Chase J Bond S Skill-mix changes and work intensification in nursing Work Employment and Society 2000 14 3 541 55

[B126] Atkinson J Manpower strategies for flexible organizations Personnel Management 1984 16 28 31

[B127] Sibbald B Shen J McBride A Zafar R Grimshaw D Changing skill mix in the NHS: A review commissioned by the human resource directorate of the Department of Health to support the NHS Changing Workforce Programme 2002 Manchester: UMIST

[B128] Hooker R Cawley J Physician assistants in American medicine 2003 2 New York: Churchill Livingstone

[B129] McPherson K Kersten P George S Lattimer V Ellis B Breton A Kaur D Frampton G Extended roles for allied health professionals in the NHS 2004 UK: National Co-ordinating Centre for NHS Service Delivery and Organisation R & D (NCCSDO) 10.1258/135581906778476544 17018199

[B130] Burns T Millar E Garland C Kendrick T Chisholm B Ross F Randomized controlled trial of teaching practice nurses to carry out structured assessments of patients receiving depot anti-psychotic injections Br J Gen Pract 1998 48 437 1845 8 10198505 PMC1313291

[B131] Crosland A Kai J "They think they can talk to nurses": practice nurses' views of their roles in caring for mental health problems Br J Gen Pract 1998 48 432 1383 6 9800394 PMC1313129

[B132] Kupshik GA Fisher CR Assisted bibliotherapy: effective, efficient treatment for moderate anxiety problems Br J Gen Pract 1999 49 438 47 8 10622018 PMC1313319

[B133] Mann AH Blizard R Murray J Smith JA Botega N MacDonald E Wilkinson G An evaluation of practice nurses working with general practitioners to treat people with depression Br J Gen Pract 1998 48 426 875 9 9604408 PMC1409896

[B134] Department of Health The NHS Plan: a plan for investment, a plan for reform 2000 London: Department of Health

[B135] Department of Health Meeting the Challenge: A strategy for the allied health professions 2000 London: Department of Health

[B136] LeRoy L Solkowitz S Case Study 16: The Costs and Effectiveness of Nurse Practitioners 1981 Washington, DC: Office of Technology Assessment

[B137] Horrocks S Anderson E Salisbury C Systematic review of whether nurse practitioners working in primary care provide equivalent care to doctors BMJ 2002 324 819 823 10.1136/bmj.324.7341.819 11934775 PMC100791

[B138] Hodson DM The evolving role of advanced practice nurses in surgery AORN Journal 1998 67 998 1009 10.1016/S0001-2092(06)62624-0 9592606

[B139] Brown J The handmaiden's tale ... practice nurses ... support workers Practice Nurse 1995 10 4 257 260

[B140] Brown SA Grimes DE A meta-analysis of nurse practitioners and nurse midwives in primary care Nursing Research 1995 44 332 339 10.1097/00006199-199511000-00003 7501486

[B141] Knedle-Murray ME Oakley D Wheeler JRC Peterson B Production process substitution in maternity care: issues of cost, quality, and outcomes by nurse-midwives and physician providers Med Care Rev 1993 50 1 81 112 10.1177/002570879305000106 10125119

[B142] Bostrom J Zimmerman J Restructuring nursing for acompetitive health care environment Nursing Economics 1993 11 35 41 54 8455729

[B143] Powers P Dickey C Ford A Evaluating an RN/co-worker model J Nurs Adm 1990 20 11 15 2313369

[B144] Garfink C Kirby KK Bachman SS The University Hospital nurse extender program: Part IV, What have we learned J Nurs Adm 1991 21 4 26 31 2010827

[B145] Seale C Anderson A Kinnersley P Comparison of GP and nurse practitioner consultations: an observational study Br J Gen Pract 2005 55 938 943 16378563 PMC1570503

[B146] Venning P Durie A Roland M Roberst C Leese B Randomised control trial comparing cost effectiveness of general practitioners and nurse practitioners in primary care BMJ 2000 320 1048 53 10.1136/bmj.320.7241.1048 10764367 PMC27348

[B147] Edwards M The health care assistant: usurper of nursing British Journal of Community Health Nursing 1997 10 490 4

[B148] Orne RM Garland D O'Hara M Perfetto L Stielau J Caught in the cross fire of change: nurses' experience with unlicensed assistive personnel Applied Nursing Research 1998 11 101 10 10.1016/S0897-1897(98)80092-X 9757609

[B149] Humphrey C Ehrich K Kelly B Policies affecting human resource management in the NHS and their implications for continuity of care 2002 London: National Co-ordinating Centre for NHS Service Delivery and Organisation R & D

[B150] Richardson G Maynard A Fewer doctors? More nurses? A review of the knowledge base of doctor/nurse substitution. Discussion Paper 135 1995 University of York

[B151] Dale J Crouch R Lloyd D Primary care: nurse-led telephone triage and advice out-of-hours Nurs Stand 1998 12 47 41 5 9752159

[B152] Gallagher M Huddart T Henderson B Telephone triage of acute illness by a practice nurse in general practice: outcomes of care Br J Gen Pract 1998 48 1141 1145 9667088 PMC1410031

[B153] Jones K Gilbert P Little J Wilkinson K Nurse triage for house call requests in a Tyneside general practice: patients' views and effect on doctor workload Br J Gen Pract 1998 48 1303 1306 9747546 PMC1410152

[B154] Wanless D Securing our future health: taking a long-term view 2002 London: HM Treasury Public Enquiry Unit

[B155] Poole J A role change for auxiliaries Nursing Times 1998 94 44 61 9919259

[B156] Jeffreys L Clarke A Koperski M Practice Nurses' workload and consultations Br J Gen Pract 1995 45 415 418 7576846 PMC1239335

[B157] Haggerty JL Reid RJ Freeman GK Starfield BH Adair CE McKendry R Continuity of care: a multidisciplinary review BMJ 2003 327 1219 1221 10.1136/bmj.327.7425.1219 14630762 PMC274066

[B158] Schers H Ven C van de Hoogen H van den Grol R Bosch W van den Family medicine trainees still value continuity of care Fam Med 2004 36 51 54 14710330

[B159] Laurant MG Hermens RP Braspenning JC Sibbald B Grol RP Impact of nurse practitioners on workload of general practitioners: randomised controlled trial BMJ 2004 328 7445 927 10.1136/bmj.38041.493519.EE 15069024 PMC390208

[B160] Charles-Jones H Latimer J May C Transforming general practice: the redistribution of medical work in primary care Sociol Health Illn 2003 25 71 92 10.1111/1467-9566.t01-1-00325 14498945

[B161] Calpin-Davies PJ Doctor-nurse substitution: the workforce equation J Nurs Manag 1999 7 71 79 10.1046/j.1365-2834.1999.00113.x 10373846

[B162] Buchan J Calman L Skill-mix and policy change in the health workforce: Nurses in advanced roles 2005 Paris: OECD

[B163] Richardson G Identifying, evaluating and implementing cost-effective skill mix J Nurs Manag 1999 7 5 265 70 10.1046/j.1365-2834.1999.00137.x 10786545

[B164] Spilsbury K Meyer J Use, misuse and non-use of health care assistants: understanding the work of health care assistants in a hospital setting J Nurs Manag 2004 12 411 418 10.1111/j.1365-2834.2004.00515.x 15509270

[B165] Savitz LA Jones CB Bernard S Quality indicators sensitive to nurse staffing in acute care settings Advances in Patient Safety: from Research to Implementation 2005 4 375 385 21250026

[B166] Ayre TC Gerdtz MF Parker J Nelson S Nursing skill mix and outcomes: a Singapore perspective Int Nurs Rev 2007 54 1 56 62 10.1111/j.1466-7657.2007.00518.x 17305958

[B167] Martin-Alcazar F Romero-Fernandes PM Sanchez-Gardey G Strategic human resource management: integrating the universalistic, contingent, configurational and contextual perspectives Int J of human resource management 2005 16 5 633 659

[B168] Lengnick-Hall CA Lengnick-Hall ML Strategic human resource management: a review of the literature and a proposed typology The Academy of Management Review 1988 13 3 454 70 10.2307/258092

[B169] Schuler RS Strategic human resource management: linking people with the needs of the business Organizational Dynamics 1992 21 19 32 10.1016/0090-2616(92)90083-Y

[B170] Wright PM McMahan GC Theoretical perspectives for strategic human resource management Journal of Management 1992 18 2 295 320 10.1177/014920639201800205

[B171] Wright PM Snell SA Toward an integrative view of strategic human resource management Human Resource Management Review 1991 1 203 25 10.1016/1053-4822(91)90015-5

[B172] Jackson SE Schuler RS Understanding Human Resource Management in the Context of Organizations and their Environments Annual Review of Psychology 1995 46 237 64 10.1146/annurev.ps.46.020195.001321 19245335

[B173] Martínez J Martineau T Rethinking human resources: an agenda for the millennium Health Policy and Planning 1998 13 345 358 10.1093/heapol/13.4.345 10346027

[B174] Institute of Medicine Nursing staff in hospitals and nursing homes: is it adequate? 1996 Washington, DC: National Academy Press 25121200

[B175] Aiken LH Smith HL Lake ET Lower Medicare mortality among a set of hospitals known for good nursing care Med Care 1994 32 8 771 87 10.1097/00005650-199408000-00002 8057694

[B176] Mathieu JE Marks MA Zaccaro SJ Anderson N, Ones DS, Sinangil HK, Viswesvaran C Multiteam systems Handbook of industrial, work and organizational psychology 2001 2 London: Sage 289 313

[B177] Hackman JR Leading teams: Setting the stage for great performances 2002 Boston, MA: Harvard Business School Press

[B178] Hackman JR Groups that work (and those that don't): Creating conditions for effective teamwork 1990 San Francisco, CA: Jossey-Bass

[B179] Haward R Amir Z Borrill C Dawson J Scully J West M Sainsbury R The impact of constitution, new cancer workload, and methods of operation on effectiveness Br J Cancer 2003 89 1 15 22 10.1038/sj.bjc.6601073 12838294 PMC2394209

[B180] Curley C McEachern JE Speroff T A firm trial of interdisciplinary rounds on the inpatient medical wards Med Care 1998 36 Suppl 8 AS4 12 10.1097/00005650-199808001-00002 9708578

[B181] Becker BE Gerhart B The impact of human resource management on organizational performance: progress and prospects The Academy of Management Journal 1996 39 4 779 801 10.2307/256712

[B182] Whitfield K Poole M Organising employment for high performance: theories, evidence and policy Organization Studies 1997 18 5 745 764 10.1177/017084069701800502

[B183] Ramsay H Scholarios D Harley B Employees and high-performance work systems British Journal of Industrial Relations 2000 34 4 501 532 10.1111/1467-8543.00178

[B184] Bowen DE Ostroff C Understanding HRM-firm performance linkages: the role of the "strength" of the HRM system Academy of Management Review 2004 29 2 203 221

[B185] Collins CJ Clark KD Strategic human resource practices, top management team social networks, and firm performance: the role of human resource practices in creating organizational competitive advantage Academy of Management Journal 2003 46 6 740 751

[B186] Tsui AS Pearce JL Porter LW Tripoli AM Alternative approaches to the employee -organization relationship: does investment in employees pay off Academy of Management Journal 1997 40 5 1089 1121 10.2307/256928

[B187] Griffin MA Mathieu JE Modeling organisational processes across hierarchical levels: climate, leadership and group processes in work groups Journal of Organisational Behavior 1997 18 731 744 10.1002/(SICI)1099-1379(199711)18:6<731::AID-JOB814>3.0.CO;2-G

[B188] Pritchard RD Karasek BW The effects of organisational climate on managerial job performance and satisfaction Organisational Behavior and Human Performance 1973 9 126 146 10.1016/0030-5073(73)90042-1

[B189] World Health Organization Working together for health - the World Health Report 2006. Geneva 2006

[B190] Stordeur S D'hoore W Vandenberghe C Leadership, organizational stress and emotional exhaustion among nursing hospital staff Journal of Advanced Nursing 2001 35 533 542 10.1046/j.1365-2648.2001.01885.x 11529953

[B191] Beaulieu D Horrigan DR Putting smart money to work for quality improvement Health Serv Res 2005 40 1318 1334 10.1111/j.1475-6773.2005.00414.x 16174136 PMC1361200

[B192] Michie S West M Managing people and performance: An evidence based framework applied to health service organisations. International Journal of Management Reviews 2004 5/6 2 91 111

[B193] Franco LM Bennett S Kanfer R Health sector reform and public sector health worker motivation: a conceptual framework Soc Sci Med 2002 54 8 1255 66 10.1016/S0277-9536(01)00094-6 11989961

[B194] Ingersoll GL Kirsch JC Merk SE Lightfoot J Relationship of organizational culture and readiness for change to employee commitment to the organization J Nurs Adm 2000 30 1 11 20 10.1097/00005110-200001000-00004 10650431

[B195] McClure ML Hinshaw AS Magnet hospitals revisited: attraction and retention of professional nurses 2002 Washington DC: Amercian Nurses Association

[B196] Brimberry R Vaccination of high-risk patients for influenza: a comparison of telephone and mail reminder methods Journal of Family Practice 1988 26 397 400 3282026

[B197] Friedman RH Kazis LE Jette A Smith MB Stollerman J Torgerson J Carey K A telecommunications system for monitoring and counseling patients with hypertension: impact on medication adherence and blood pressure control Am J Hypertens 1996 4 285 292 10.1016/0895-7061(95)00353-3 8722429

[B198] Shortell SM Zimmerman JE Rousseau DM Gillies RR Wagner DP Draper EA Knaus WA Duffy J The performance of intensive care units: Does good management make a difference? Med Care 1994 32 5 508 25 10.1097/00005650-199405000-00009 8182978

[B199] Rousseau DM Psychological contracts in organizations: understanding written and unwritten agreements 1995 Thousand Oaks, CA: Sage

[B200] Huselid M The Impact of Human Resource Management Practices on Turnover, Productivity, and Corporate Financial Performance Academy of Management Journal 1995 38 3 972 991 10.2307/256741

[B201] Ichniowski C Shaw K Prenushi G The effects of human resource practices on productivity: a study of steel finishing lines American Economic Review 1997 87 3 291 313

[B202] MacDuffie JP Human resource bundles and manufacturing performance: organizational logic and flexible production systems in the world auto industry Industrial and Labor Relations Review 1995 48 2 197 221 10.2307/2524483

[B203] Normand CEM Malek M, Vacani P, Rasquinha J, et al Changing patterns of care - the challenges for health care professions and professionals Managerial Issues in the Reformed NHS 1993 Chichester: John Wiley and Sons 237 245

[B204] Hunter DJ The changing roles of health care personnel in health and health care management Soc Sci Med 1996 43 5 799 808 10.1016/0277-9536(96)00125-6 8870145

[B205] Kirby MJL Keon W Why Competition Is Essential in the Delivery of Publicly Funded Health Care Services Policy Matters 2004 5 8 1 32

[B206] Tarrant, F and Associates Literature review report: supports, barriers and impediments to practice. Ottawa: Canadian Nurses Association 2005

[B207] Priest A What's ailing our nurses? A discussion of the major issues affecting nursing human resources in Canada 2006 Ottawa: Canadian Health Services Research Foundation

[B208] Hall P Weaver L Interdisciplinary education and teamwork: a long and winding road Med Educ 2001 35 9 867 75 10.1046/j.1365-2923.2001.00919.x 11555225

[B209] Crozier K Interprofessional education in maternity care: shared. learning for women-centred care International Journal of Sociology and Social Policy 2003 23 4-5 123 138 10.1108/01443330310790543

[B210] Richardson G Maynard A Cullum N Kindig D Skill mix changes: substitution or service development? Health Policy 1998 45 2 119 32 10.1016/S0168-8510(98)00038-4 10186223

[B211] Dubois C-A McKee M Cross-national comparisons of human resources for health - What can we learn? Journal of Health Economics, Policy and Law 2006 1 59 78 10.1017/S1744133105001027 18634703

[B212] Hollander MJ Anderson M Béland F Havens B Keefe J Lawrence W Parent K Ritter R The identification and analysis of incentives and disincentives and cost-effectiveness of various funding approaches for continuing care: Final report 2000 Victoria, BC: Hollander Analytical Services

[B213] Health Council of Canada Modernizing the Management of Health Human Resources in Canada: Identifying Areas for Accelerated Change. Report from a National Summit. Toronto 2005

